# Functional polymorphism rs3783553 in the 3’-untranslated region of *IL-1A* increased the risk of ischemic stroke: A case-control study

**DOI:** 10.1097/MD.0000000000008522

**Published:** 2017-11-17

**Authors:** Peng Wang, Qian He, Chen Liu, Shi Zhen He, Shou Yan Zhu, Ying Wen Li, Wei Su, Shu Tian Xiang, Bo Zhao

**Affiliations:** Department of Radiology, the Second People's Hospital of Yunnan Province, Kunming, Yunnan, China.

**Keywords:** interleukin-1α, ischemic stroke, plasma, polymorphism

## Abstract

Accumulating evidence indicates interleukin-1 (IL-1) is a critical mediator of inflammatory responses in ischemic stroke (IS). The aim of this study was to investigate whether rs3783553 in the 3’-untranslated region of *IL-1A* was associated with the risk of IS. In this hospital-based case-control study, we genotyped the rs3783553 using polymerase chain reaction in 316 patients with IS and 332 age, sex, and ethnicity-matched controls. Plasma level of IL-1α was measured by enzyme-linked immunosorbent assay. The relative luciferase activities were measured by the Dual Luciferase assay system. The presence of ins/ins genotype was associated with higher odds ratios (ORs) of IS compared with del/del genotype (ins/ins vs del/del: adjusted OR 1.77, 95% confidence interval [CI] 1.06–2.98; recessive model: adjusted OR 1.69, 95% CI 1.06–2.70). The higher risk of IS was also observed in allele comparison (adjusted OR 1.29, 95% CI 1.00–1.65). Multivariate logistic regression analysis showed that age, hypertension, total cholesterol, triglyceride, low-density lipoprotein, and rs3783553ins/ins genotypes were independent risk factors for IS. Plasma level of IL-1α was higher among IS patients compared with controls (*P* = .03). Notably, IS patients with the TTCA/TTCA genotype had a higher level of IL-1α compared with those with the del/del genotype (*P* = .01). Luciferase reporter assay showed that the vector containing the TTCA del allele exhibited a reduced transcriptional activity in the presence of miR-122 and miR-378. These findings indicate that *IL-1A* rs3783553 ins/ins genotype may increase the susceptibility to IS, possibly by interrupting the binding site of miR-122 and miR-378.

## 1 Introduction

As the second leading cause of death and a major cause of functional disability, stroke remains a serious health problem worldwide.^[1]^ Ischemic stroke (IS), the most common type of stroke, accounts for 43% to 79% of all such events.^[2]^ IS is an extremely complex disorder with multifactors involved.^[3–7]^ Epidemiology studies in family history and twins showed that genetics is related to the pathogenesis of IS.^[3–5]^ Apart from genetic factors, inflammation is recognized as a prominent contributor to IS.^[6,7]^

Interleukin-1 (IL-1), a proinflammatory cytokine, plays a crucial role in IS incidence.^[8–10]^ IL-1 contains 3 family members: IL-1α, IL-1β, and IL-1 receptor antagonist (IL-1Ra). In animal models, IL-1α was observed to be up-regulated after occlusion of middle cerebral artery, inducing proliferation, migration, and angiogenesis in brain endothelial cells.^[8,11–13]^ All of these responses can be blocked by IL-1RA.^[8]^ In humans, mRNA levels of both *IL-1β* and *IL-1RA* were found to be higher in atherosclerotic arteries.^[14]^ It is well known that intracranial atherosclerosis is a risk factor for IS.^[15]^ These findings strongly suggest that IL-1 is implicated in the pathophysiology of IS.

In 2009, Gao et al^[16]^ reported a functional TTCA insertion/deletion polymorphism (rs3783553) in the 3’-untranslated region (UTR) of *IL-1A*, which can alter micro (miR)-122 and miR-378 mediated regulation of IL-1α expression. miR-122 was down-regulated in IS, representing a potential biomarker and target for the prevention and treatment of IS.^[17,18]^ Elevating miR-122 can improve stroke outcomes via down-regulating its target genes.^[19]^ To date, *IL-1A* rs3783553 has been investigated in varieties of human diseases, including hepatocellular carcinoma,^[16]^ papillary thyroid carcinoma,^[20]^ endometriosis,^[21,22]^ preeclampsia,^[23]^ osteoarthritis,^[24]^ hepatitis B,^[25]^ and nonalcoholic fatty liver disease.^[26]^ However, no data about the relationship between *IL-1A* rs3783553 and the risk of IS were reported. In this study, we proceeded an investigation to identify whether the *IL-1A* rs3783553 was a risk factor for IS in a Chinese Han population. Additionally, whether the polymorphism influenced plasma IL-1α expression and luciferase activity was also examined.

## 2 Materials and methods

### 2.1 Study population

The study population consisted of Chinese Han subjects enrolled in a hospital-based case-control study conducted in the Second People's Hospital of Yunnan Province. Quanto software (version 1.2.3) was used to estimate the sample size. When we set the *R*_g_ = 1.60 and statistical power = 80%, the required sample size is 305 under a dominant model. In this study, 328 cases and 340 controls were collected to analyze the association between the rs3783553 and IS risk. Subject recruitment began on October 10, 2011 and continued consecutively on December 21, 2015. During the time period, 328 IS patients agreed to participate in the study. IS was diagnosed by clinical manifestations and confirmed by computed tomography scans and magnetic resonance imaging. IS subtypes were classified using the criteria of Trial of Org 10172 in Acute Stroke Treatment as previously described.^[27]^ We excluded patients if they had hemorrhagic stroke, subarachnoid hemorrhage, and major trauma, cardiac, hepatic, and renal disorders. Hypertension was defined as a systolic and/or a diastolic blood pressure consistently higher than 139 mm Hg and/or 89 mm Hg.^[28]^ Diabetes mellitus was defined as 2 fasting glucose measurements above 7.0 mmol/L. During the same period, 340 volunteers who came to the hospital for physical examination were selected as controls according to the following criteria: (1) Chinese Han ethnicity; (2) healthy individuals confirmed by routine physical examination; (3) living in the same area. The controls were frequency-matched to cases based on age, sex, and ethnicity. Clinical data collection in this study involved some variables relevant to IS, such as age, sex, living area, ethnicity, hypertension, diabetes, total cholesterol (TCH), triglyceride (TG), high-density lipoprotein cholesterol (HDL-C), and low-density lipoprotein cholesterol (LDL-C). The study protocol was approved by the Institutional Review Board of the Second People's Hospital of Yunnan Province, and all participants provided informed consent.

### 2.2 Genotyping

We selected the rs3783553 according to the following criteria: (1) rs3783553 located in the 3’-UTR of *IL-1A*, with a function of disrupting the binding site of miR-122 and miR-378^[16,29]^; (2) IL-1α is a critical mediator of inflammatory responses in IS; and (3) miR-122 may be used as a biomarker in IS, involving in the process of postischemic neuronal damage.^[16–18]^ From each subject, 2 to 3 mL venous blood sample was collected. After centrifugation at 1600 rpm and 4°C for 10 minutes, plasma was aliquoted and stored at −80°C until analysis. White blood cells were separated to extract genomic DNA using a commercial kit (Tiangen, Beijing, China). *IL-1A* rs3783553 was genotyped by polymerase chain reaction (PCR). The primers and PCR conditions were described previously.^[20]^ The genotyping results were confirmed by DNA sequencing.

### 2.3 Enzyme-linked immunosorbent assay

Fifty-two cases and controls were randomly selected to measure plasma level of IL-1α using enzyme-linked immunosorbent assay according to the manufacturer's instructions (RayBiotech, Norcross, GA). The experiments were done in duplicate.

### 2.4 *IL1A* 3’ UTR luciferase reporter assay

The luciferase plasmids containing the full length of *IL-1A* 3’UTR were constructed as described previously.^[16]^ Briefly, human genomic DNA samples of TTCA/TTCA insertion and −/− deletion were used as templates to amplify *IL-1A* 3’UTR using the primers: 5’-GATCTCTAGAGTCTGGAGTCTCACTTGTCTCACTTGTG-3’ (forward) and 5’-CATGGATCCGTCAGAGAATTTTGTTGCAAGCTTTATTTAG-3’ (reverse). After Sanger sequencing, the plasmids were inserted into pRL-SV40 (Promega, Madison, WI).

The HEK293 cells were cultured in Dulbecco modified Eagle medium with 10% fetal bovine serum at a 37°C incubator supplemented with 5% CO_2_. Cells were seeded in 24-well plates (1 × 10^5^ cells/well), followed by transfection with 500 ng pRL vector, 50 ng pGL3 control vector, in combination with different concentrations of miR-122 or miR-378 (1, 10, and 100 pmol). At 24 hours after transfection, luciferase activities were measured by the Dual Luciferase assay system (Promega).

### 2.5 Statistical analysis

The statistical analysis was done using SPSS 19.0 software (SPSS, Chicago, IL). Quanto software (version 1.2.3) was used to estimate the sample size. The rs3783553 genotypic and allelic frequencies were determined by direct counting. The genotype distribution was checked for deviation from Hardy-Weinberg equilibrium (HWE) by chi-square test. Case-control comparisons for categorical or continuous data were performed using chi-square or Student *t* test, respectively. The association between rs3783553 and IS was assessed by computing odds ratios (ORs) and 95% confidence intervals (CIs). ORs were adjusted based on age, sex, hypertension, and diabetes mellitus using logistic regression analyses. Multivariate logistic regression analysis was performed to identify independent risk factors for IS. Plasma level of IL-1α was expressed as mean ± standard deviation (SD). The statistical significance of IL-1α level in IS patients and controls was estimated using Mann-Whitney *U* test. Stratification analysis and gene luciferase reporter activities were measured by 1-way analysis of variance (ANOVA). Values of *P* < .05 were considered as significant.

### 2.6 Ethical approval and informed consent

All procedures performed in studies involving human participants were in accordance with the Institutional Review Board of the Second People's Hospital of Yunnan Province and with the 1964 Helsinki declaration and its later amendments or comparable ethical standards.

Informed consent was obtained from all individual participants included in the study.

## 3 Results

Due to DNA quality, 316 cases and 332 controls were genotyped successfully. We deleted the samples without successful genotyping data. The general characteristics of IS patients and controls are shown in Table 1. The mean age of cases and controls was 59.72 ± 10.79 and 58.43 ± 10.51 years, respectively. The sex distribution among IS patients and controls was similar, with male/female ratio of about 2:1, suggesting that age and sex were comparable. The IS patients had a higher frequency of hypertension and diabetes mellitus than the controls. Levels of TCH, TG, and LDL-C were significantly higher in IS group compared with control group.

**Table 1:**
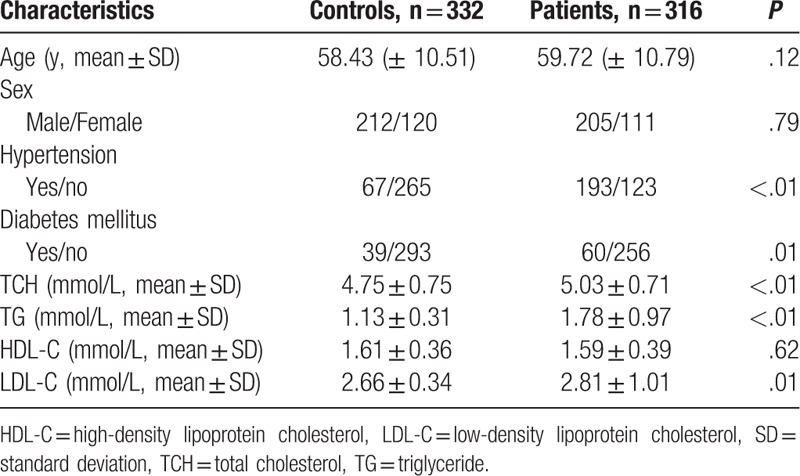
Characteristics of the study population.

Genotype and allele frequencies of rs3783553 in IS patients and controls are summarized in Table 2. The genotype distributions among controls were in HWE (*χ*^2^ = 0.03, *P* = .86). The presence of ins/ins (TTCA/TTCA) genotype was associated with a higher OR of IS compared with del/del (−/−) genotype (ins/ins vs del/del: adjusted OR 1.77, 95% CI 1.06–2.98; recessive model: adjusted OR 1.69, 95% CI 1.06–2.70). The higher risk of IS was also observed in allele comparison (ins vs del: adjusted OR 1.29, 95% CI 1.00–1.65). In stratification analysis according to TCH, TG, HDL-C, and LDL-C, no association was observed between rs3783553 and these parameters of IS patients (Table 3).

**Table 2:**
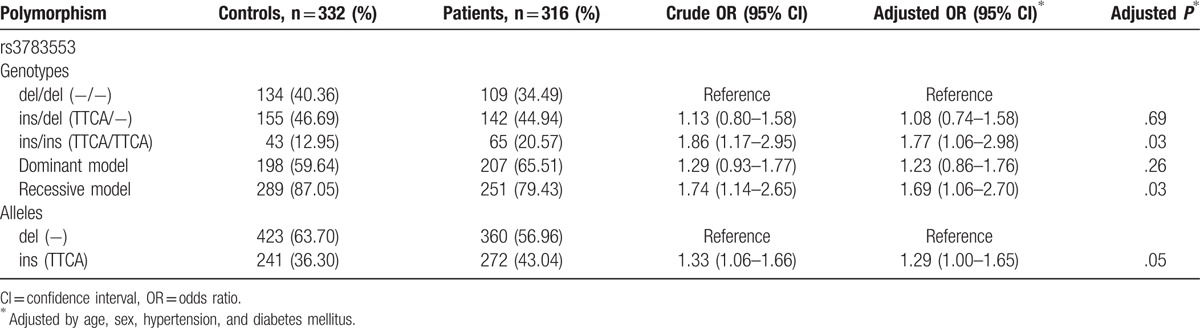
Association between rs3783553 and risk of ischemic stroke.

**Table 3:**
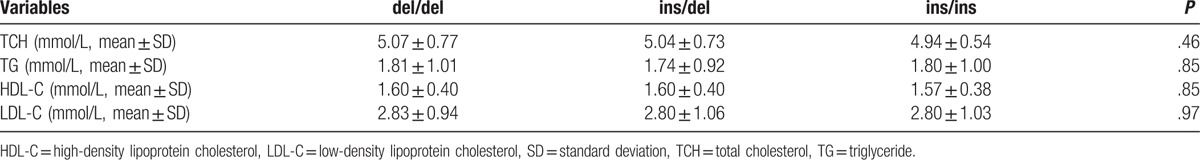
Association between rs3783553 and clinical characteristics of ischemic stroke patients.

We then used multivariate logistic regression analysis to identify independent risk factors for IS. As shown in Table 4, the risk factors were age (OR 1.03; 95% CI 1.01–1.05), hypertension (OR 9.01; 95% CI 6.02–13.51), TCH (OR 1.47, 95% CI 1.15–1.85), TG (OR 5.26, 95% CI 3.57–7.69), LDL (OR 0.57, 95% CI 0.43–0.76), and rs3783553ins/ins genotype (OR 1.19, 95% CI 1.01–1.41).

**Table 4:**
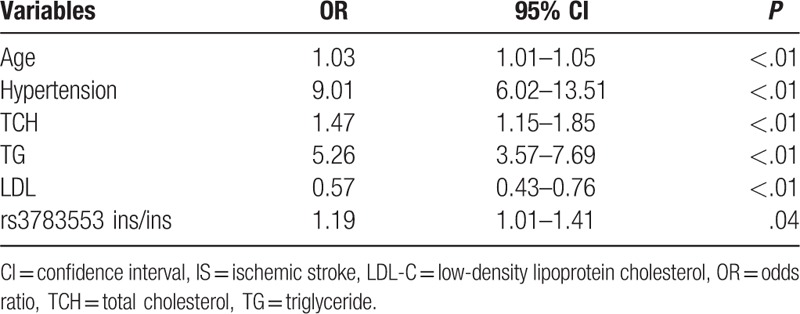
Multivariate logistic regression analysis of independent risk factors for IS.

**Figure 1:**
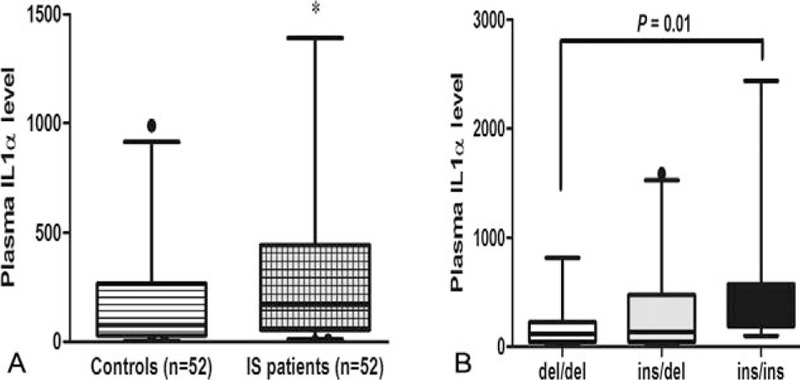
(A) Plasma level of interleukin (IL)-1α in patients with ischemic stroke than controls (^∗^*P* < .05). (B) The relationship between the rs3783553 genotype and plasma level of IL-1α in patients with ischemic stroke. Data are presented as mean ± SD.

Plasma level of IL-1α was higher among patients with IS than controls (mean ± SD: 316.3 ± 434.6 vs 212.7 ± 328.3 pg/mL; *P* = .03) (Fig. 1A). We further evaluated whether the rs3783553 affected plasma level of IL-1α. Of the 52 serum samples, 19 were del/del (−/−) genotype, 23 were ins/del (+/−) genotype, and 10 were ins/ins (TTCA/TTCA) genotype. IS patients with the TTCA/TTCA genotype had a higher level of IL-1α compared with those with the del/del genotype (*P* = .01) (Fig. 1B).

**Figure 2:**
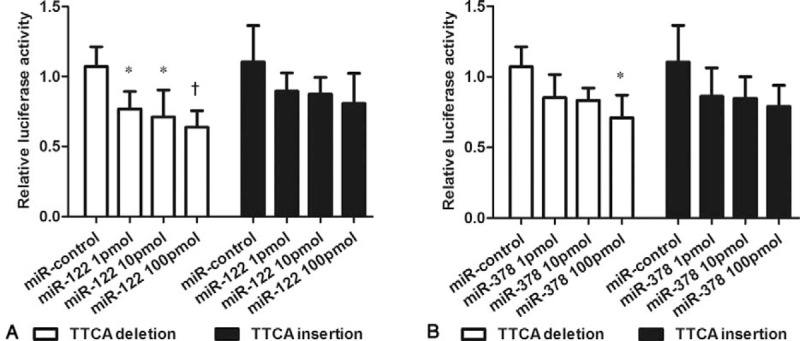
The binding of the rs3783533 with 3’UTR of *IL-1A* in the presence of miR-122 (A) or miR-378 (B) in HEK293 cells. The relative luciferase activities were measured by the Dual Luciferase assay system. Data are presented as mean ± SD. ^∗^*P* < .05, ^†^*P* < .01.

Previous studies have shown the rs3783553 can alter the binding of both miR-122 and miR-378 in hepatocellular carcinoma and gastric cancer cell lines.^[16,29]^ In this study, we constructed plasmids to examine whether the polymorphism can affect the binding of miR-122 and miR-378 with the 3’UTR of *IL-1A* in HEK293 cells. As shown in Fig. 2A, compared with the control, the vector containing the TTCA del allele showed a reduced transcriptional activity in the presence of miR-122 in a dose-dependent manner (*P* < .05). Although the vector containing the TTCA ins allele had a decreased trend of luciferase activity, no significant difference was observed. Similar results were also found in the presence of 100 pmol, but not 1 and 10 pmol miR-378 (Fig. 2B).

## 4 Discussion

In this hospital-based case-control study, we firstly investigated the association between the *IL-1A* rs3783553 and risk of IS in a Chinese Han population. We found that the ins/ins genotype was associated with an increased risk of IS. Multivariate logistic regression analysis showed that age, hypertension, TCH, TG, LDL, and rs3783553ins/ins genotype were independent risk factors for IS. Genotype-phenotype analysis showed that IS patients with the TTCA/TTCA genotype had a higher plasma level of IL-1α compared with those with the del/del genotype. Luciferase reporter assay showed that the vector containing the TTCA del allele exhibited a reduced transcriptional activity in the presence of miR-122 and miR-378.

Inflammation has been recognized as a major contributor to the worsening of IS.^[6,7]^ Increasing production of several proinflammatory cytokines, including IL-1, has been demonstrated in plasma of stroke patients.^[30]^ After cerebral ischemia, both IL-1α and IL-1β express in microglia, astrocytes, and endothelial cells, inducing activation of endothelial cells and astrocytes and promoting formation of tube-like structure that is a key hallmark of angiogenesis.^[8,31]^ Knockout IL-1α and IL-1β in mice can reduce ischemic damage caused by middle cerebral artery occlusion.^[13]^ Deletion of blood-derived IL-1 can improve neurological outcome after experimental ischemic brain injury.^[9]^ These findings indicate that IL-1 plays a crucial role and maybe a potential target for the therapy of IS.

Previously, Gao et al^[16]^ reported that the homozygote ins/ins genotype of rs3783553 was associated with a significantly reduced risk of hepatocellular carcinoma. Subsequent studies investigated the association between the rs3783553 and a series of inflammatory-related diseases. Chen et al^[23]^ reported that the rs3783553 TTCA insertion allele reduced the individual's susceptibility for preeclampsia. The decreased risk was also observed in endometriosis.^[21,22]^ Moreover, the decreased-risk genotype was reported to relate to an increasing body mass index, decreasing HDL-C, and low level of serum IL-1α in Egyptian patients with nonalcoholic fatty liver disease.^[26]^ Yang et al^[24]^ reported that the rs3783553 TTCA del allele was associated with an elevated risk for osteoarthritis and disease severity. The elevated risk was also observed to be related to hepatitis B among the Asian population.^[25]^ In this study, we found that the ins/ins (TTCA/TTCA) genotype was associated with an increased risk of IS. IL-1α is a critical mediator of inflammatory responses in IS as mentioned above. miR-122 may be used as a biomarker in IS, involving in the process of postischemic neuronal damage.^[16–18]^ Moreover, rs3783553 is located in the 3’-UTR of *IL-1A*, with a function of disrupting the binding site of miR-122 and miR-378,^[16,29]^ and thus the positive result in this study seems to be reasonable.

Apart from the rs3783553 in the 3’-UTR of *IL-1A*, *IL-1A*-889C/T and *IL-1B* -511C/T have been studied extensively. Although the *IL-1A* -889 T allele was reported to be a risk factor for IS in 3 independent studies,^[32–34]^ meta-analysis showed that the polymorphism was not associated with the risk of IS.^[35,36]^ As for the *IL-1B*-511C/T, conflicting results have been obtained in different research groups. Dziedzic et al^[37]^ reported that the *IL-1B*-511TT genotype had a 2.40-fold increased risk of small vessel disease stroke in a Polish population. However, Zhang et al^[38]^ failed to find any association between the *IL-1B*-511C/T and IS risk in a Chinese population. Subsequent meta-analysis supported the negative result.^[39]^ These findings suggest that the rs3783553, but not *IL-1A*-889C/T and *IL-1B*-511C/T, may be a risk factor for IS. Further validation analyses are necessary to confirm the results.

Regarding the potential mechanism, we postulated that the *IL-1A* rs3783553 may influence IL-1α expression and eventually result in individuals’ susceptibility to IS. The *IL-1A* rs3783553 was predicted to have different binding affinity to miR-122 and miR-378, which modulate the expression of IL-1α in a negative manner.^[16]^ Specifically, the *IL-1A* rs3783553 TTCA del allele can bind tightly to miR-122 and miR-378, whereas the TTCA ins allele cannot, causing up-regulation of IL-1α.^[16,24]^ Luciferase reporter assay in this study showed that exogenous overexpression of miR-122 or miR-178 displayed a decreased luciferase activity. We then measured plasma level of IL-1α to examine whether the rs3783553 can influence the expression of IL-1α. We found that the ins/ins exhibited a higher level of plasma IL-1α. Taken together, these findings indicate that the TTCA ins allele disrupted the binding of miR-122 and miR-378 with 3’UTR of *IL-1A*, allowing up-regulated level of IL-1α, and finally resulting in increasing risk of IS.

The strengths of this study include consistent reporting of HWE in controls and the use of logistic regression to adjust ORs. The study, however, has some limitations. The results were generated from Han Chinese population, which cannot be applicable to other ethnicities. Follow-up data were not available in this study, which prevents our further survival analysis. Population-based and long-term follow-up studies in different ethnicities are warranted to confirm our findings.

## 5 Conclusions

In conclusion, to the best of our knowledge, this is the first study to examine the association of rs3783553 in the 3’-UTR of *IL-1A* with the risk of IS in a Chinese population. The results of the present study revealed that the rs3783553 ins/ins genotype significantly increased the risk of IS, possibly by disrupting the binding of miR-122 and miR-378 and leading to higher expression of IL-1α. These findings suggest that rs3783553 may be used as a promising biomarker for IS. A prospective validation of this finding is necessary in a larger cohort to obtain much more clear information about the influence of *IL-1A* rs3783553 on IS risk.
